# The effect of a water-soluble β-glucan on intestinal immunity and microbiota in LPS-challenged piglets

**DOI:** 10.3389/fvets.2025.1533872

**Published:** 2025-03-10

**Authors:** Yuliang Wu, Yuxin Li, Mengli Chen, Juan Zhao, Xia Xiong, Chen Guang Olnood, Yundi Gao, Fei Wang, Can Peng, Miao Liu, Chunxia Huang, Jianzhong Li, Liuqin He, Huansheng Yang, Yulong Yin

**Affiliations:** ^1^Laboratory of Animal Nutrition and Human Health, College of Life Sciences, Hunan Normal University, Changsha, China; ^2^CAS Key Laboratory of Agro-Ecological Processes in Subtropical Region, Institute of Subtropical Agriculture, Hunan Province Key Laboratory of Animal Nutritional Physiology and Metabolic Process, National Engineering Laboratory for Pollution Control and Waste Utilization in Livestock and Poultry Production, Institute of Subtropical Agriculture, Chinese Academy of Sciences, Changsha, China; ^3^Sichuan Synlight Biotech Ltd., Chengdu, China; ^4^University of Chinese Academy of Sciences, Beijing, China; ^5^Changsha Medical University, Changsha, China; ^6^Key Laboratory of Mollisols Agroecology, Northeast Institute of Geography and Agroecology, Chinese Academy of Sciences, Changchun, China; ^7^Jilin Da’an Agro-Ecosystem National Observation Research Station, Changchun, China

**Keywords:** β-glucan, piglet, diarrhea, intestinal inflammation, lipopolysaccharide, intestinal microbiota

## Abstract

The intestine is the largest immune and barrier organ in the body, and diarrhea and even death during piglet development are related to dysfunction caused by intestinal barrier damage and inflammation. A water-soluble β-glucan produced by Agrobacterium ZX09 has been shown to have a beneficial effect on gastrointestinal health. The main objective of this study was to investigate whether pre-feeding β-glucan has a protective effect on LPS-induced immune stress in piglets. In this study, 24 weaned piglets (21-day-old; 6.64 ± 0.16 kg) were assigned to 4 treatments in a two × two factorial design with diet (with or without β-glucan) and immunological challenge (saline or LPS). Piglets were challenged with saline or LPS after 39 days of feeding 0 or 200 mg/kg β-glucan. The results demonstrated that β-glucan supplementation increased the average daily weight gain and daily feed intake, and decreased diarrhea rate of piglets. Intestinal inflammation symptoms and histological changes in LPS-challenged piglets were alleviated by pre-feeding of β-glucan. β-glucan supplementation reduced serum IL-1β (interleukin-1β) and NO (nitric oxide) secretion in piglets after LPS challenge (0.01 < *p* < 0.05). Supplementation with β-glucan downregulated the mRNA expression of IL-6 in piglets after LPS challenge (0.01 < *p* < 0.05). β-glucan supplementation enriched the short-chain fatty acid-producing bacteria, such as *Agathobacter* and *Subdoligranulum* (0.01 < *p* < 0.05), and increased the concentrations of propionate and butyrate (0.01 < *p* < 0.05). In conclusion, pre-feeding β-glucan can enhance piglet immunity and promote piglet growth by influencing gut microbiota composition and metabolism, and alleviate intestinal damage after LPS challenge.

## 1 Introduction

The intestinal tract is the largest immune and barrier organ in the mammalian body, and its function is to effectively prevent the invasion of foreign antigens, microorganisms, and toxins from the external environment into the interior of the organism. Piglet stress reduces feed intake, leads to intestinal dysfunction and diarrhea, and ultimately hinders growth (1, 2). The health of the piglet gut is critical to pig production, but the underlying mechanisms of intestinal epithelial cell injury currently require further investigation. Studies have shown a subtle correlation between disruption of intestinal epithelial barrier function and the development of an inflammatory state in the gastrointestinal tract (3–5). In addition, intestinal microorganisms may also be involved in the latent mechanisms of intestinal injury and repair (6, 7). In the presence of inappropriate epithelial injury and repair processes, the gut microbiota may deviate from a state of ecological imbalance or undergo migration. Therefore, it is critical to reduce intestinal inflammation or optimize the GI microbiota to maintain normal GI tract function.

Contemporary means of regulating gut health include promoting intestinal immunity and modifying the gut microbiota (8–10). β-glucan is a linear polysaccharide composed of D-glucose monomers linked by glycosidic bonds. It has been shown to selectively promote the growth or activity of intestinal bacteria, thereby impacting host health and the immune system (11, 12). Our previous study revealed that a water-soluble β-glucan (molecular weight 2000 kDa, purity 60%) from *Agrobacterium zeylanicum ZX09* improved the growth performance of weaned piglets by altering the gut microbiota (13). In addition, this β-glucan reduced obesity in mice by enriching the beneficial flora, increasing short-chain fatty acid content in the cecum (14, 15), and alleviating dextrose sodium sulfate-induced colonic inflammation (16). Thus, this water-soluble β-glucan may be beneficial for the gastrointestinal health of animals. It has potential uses as an immunomodulator and for the development of functional foods. However, the role and mechanism of β-glucan in the treatment of intestinal inflammation in piglets is unknown.

In this study, we challenged piglets with LPS (lipopolysaccharide) after 4 weeks of β-glucan supplementation and investigated the effects of β-glucan on growth, intestinal immunity, and gut microbiology of piglets after LPS challenge.

## 2 Materials and methods

### 2.1 Animal care and experimental design

The experimental design and procedures used in this study were approved by the Animal Care and Use Committee of the Institute of Subtropical Agriculture, Chinese Academy of Sciences (ISA-2021-00-20).

Twenty-four 21-day-old weaned piglets (Duroc × (Landrace × Yorkshire); 6.64 ± 0.16 kg) of similar body weight were randomly divided into four treatments in a 2 × 2 factorial design with diet (with or without β-glucan) and immunological challenge (saline or LPS). LPS was purchased from Sigma-Aldrich (*E. coli* serotype 055: B5; purity >99%; REF: L2880; St Louis, MO, USA). Following a 4-day pre-feeding period, two of the groups were provided with a basal diet, while the remaining two groups received a basal diet supplemented with 200 mg/kg of β-glucan (2,000 kDa; 50% β-glucan with 50% maltodextrin as carrier; Sichuan Synlight Biotech Ltd., Chengdu, China). Five weeks later, half of the piglets were injected intraperitoneally with LPS at 80 μg/kg body weight (17). The other half was injected with the same amount of sterilized saline. Within 4 h after the injection of LPS or normal saline, the piglets were fasted with free access to water before blood and intestinal samples were collected (18, 19) (Appendix Figure 1). The piglets were initially fed with the first-phase diet for the first 14 days, and then transitioned to the second-phase diet for the ensuing 21 days. The basal diet (Table 1) was formulated according to the National Research Council 2012 (NRC2012).

**Table 1:** Composition and nutrient level of the basal diet (air-dry basis).

	First-phase diet	Second-phase diet
Items	Content, %	Content, %
Corn 8.7%	25.00	32.42
Extruded corn	30.53	30.00
Soybean meal (46%)	8.55	10.10
Soy protein concentrate	8.00	
Fermented soybean meal		8.00
Whey powder	8.00	5.00
Fish meal	6.00	5.00
Soybean oil	2.00	2.00
Sucrose	2.00	
Glucose	5.00	3.00
Limestone powder	0.20	0.20
Calcium formate	0.60	0.60
Dicalcium phosphate	0.50	0.40
Choline chloride	0.10	0.10
Antioxidant	0.05	0.05
Citric acid	0.80	0.80
Zinc oxide	0.20	0.02
Salt	0.40	0.40
Lysine 98%	0.62	0.55
DL-Methionine	0.09	0.07
Threonine	0.25	0.20
Tryptophan 98%	0.06	0.04
Premix^1^	1.00	1.00
Antifungal Agent (Dimethyl Fumarate)	0.05	0.05
Nutrient level^2^		
DE MJ/kg	14.63	14.55
CP, %	19.01	18.16
Ca, %	0.79	0.70
TP, %	0.65	0.60
AP, %	0.42	0.38
Lys, %	1.35	1.19
Met + Cys, %	0.39	0.35
Thr, %	0.79	0.71
Trp, %	0.22	0.18

1 The premix provided the following per kilogram of the diet: VA 6450 IU, VD_3_ 2250 IU, VE 25 IU, VK 3 mg, VB_1_ 1.8 mg, VB_12_ 0.026 mg, riboflavin 8 mg, folic acid 0.9 mg, biotin 4.5 mg, niacin 24 mg, pantothenic acid 20 mg, Zn 80 mg, Fe 150 mg, Cu 10 mg, Mn 4 mg, I 0.6 mg, Se 0.5 mg, Co 0.8 mg.

2 Nutrient levels were calculated values.

### 2.2 Blood and intestinal sample collection

The piglets were given intravenous injections of 4% pentobarbital sodium solution after being given LPS or saline injections for 4 h. 15 mL of blood was collected from the jugular vein. Among them, 5 mL was placed for a routine blood test and flow cytometry after 0.5 h, and the other 10 mL was stained at 4°C for 4 h. The serum was centrifuged (3,500 *g*, 10 min) and separated in a 1.5 mL centrifuge tube and stored at −80°C. The digest was obtained from the colon. The ileum was cut longitudinally with scissors, and the digest and mucus in the intestinal cavity were washed with pre-cold saline. Furthermore, 2 cm intestinal segments were washed with pre-cold saline to remove the digesta before being fixed in a formalin fixation solution.

### 2.3 Piglet growth performance and diarrhea scores

The piglets’ health status and daily feed intake for each replicate were monitored throughout the entire experiment. For the aim of calculating ADG (average daily gain), ADFI (average daily feed intake), and F/G (feed/gain), body weight was measured every week, and feed intake was noted every day. The diarrhea rate and diarrhea score were calculated according to Equation 1 and Equation 2 below, and the piglets’ feces were scored during the test period using the following criteria: 0 for normal, solid feces; 1 for soft, looser than normal feces, mild diarrhea; 2 for moderately diarrhoeic feces; 3 for liquid, severely diarrhoeic feces (20).


(1) $$\begin{array}{l} \mathrm{Diarrhea rate}\\ =\left[\begin{array}{l} \mathrm{number of piglets with diarrhea}/\\ \left(\mathrm{number of total piglets}\times \mathrm{days of experiment}\right) \end{array}\right]\times 100\% \end{array}$$

(2) Diarrhea score=sumof fecal scores/number of total piglets

### 2.4 White blood cell counts and subsets of complete blood T cells counts

Blood was collected and placed into 5 mL Ethylenediamine Tetraacetic Acid collection tubes. Complete blood counts were performed using a Siemens hemology analyzer (Munich, Germany). Another 0.5 mL of blood sample was added to 5 mL of lysis buffer and incubated for 10 min. The reaction was stopped by diluting the lysis buffer with 10 mL of PBS. The cells were centrifuged at 4°C, and the pellet was resuspended in phosphate buffered saline. The following antibodies were used for flow cytometric analysis: anti-pig CD3 (PE, BD Biosciences, San Jose, CA, USA), anti-pig CD4 (PerCP-Cy5.5, BD Biosciences, San Jose, CA, USA), and anti-pig CD8 (Alexa 647, BD Biosciences, San Jose, CA, USA). Primary antibodies were diluted at 1:20 in PBS, after which 50 μL was added to the resuspension samples, and the mixtures were incubated for 30 min at room temperature. Cells were washed twice in PBS with 2% BSA and immediately analyzed on a FlowJo™ Software v10.8.1 (BD Life Sciences, USA). A total of 100,000 leukocytes were collected, and absolute cell counts were calculated directly by FlowJo Software.

### 2.5 Inflammatory cytokine content in plasma and ileal mucosa

The concentrations of proinflammatory cytokines in plasma and ileal mucosa were determined using porcine ELISA kits (TNF-*α* [tumor necrosis factor-α], REF: CSB-E16980; IL-1β [interleukin-1β], REF: CSB-E06782; IL-6, REF: CSB-E06786; sIgA [secretory immunoglobulin A], REF: CSB-E06786; Cusabio Biotech Co., Ltd., Hubei, China; NO [Nitric Oxide], REF: A013-2-1 Nanjing Jiancheng Bioengineering Institute, Nanjing, China).

### 2.6 Measurement of ROS in the ileum by hydroacetylene fluorescence

After being quickly frozen in liquid nitrogen, the ileal tissue was washed with cold saline and was embedded for cryosectioning. Frozen sections (8–10 μm) were stained with ROS (reactive oxygen species) staining solution at 37°C for 30 min in a light and humidified chamber. Stained sections were assessed using fluorescence microscopy. The fluorescence quantification was performed using Caseviewer on three fields per section and five sections per animal.

### 2.7 Histological evaluation

Histological evaluation was conducted according to the following procedures: The ileal and colon specimens were dehydrated, embedded in paraffin, cut into slices, and stained with hematoxylin and eosin after being fixed for 24 h. The degree of monocyte or neutrophil infiltration, histomorphology injury, intestinal epithelial cell dysplasia, or erosion was measured using Image-Pro Plus software to evaluate the morphological changes in the intestine. The histological evaluation result was divided into four grades: grade 1 (normal morphology scored 1–3); grade 2 (slight morphological injury scored 4–6); grade 3 (moderate morphological damage scored 7–9); and grade 4 (severe morphological damage scored 10–12).

### 2.8 Quantitative real-time PCR (qRT-PCR)

RNA extraction and RT-qPCR were performed according to a previous study (21). Briefly, total RNA was isolated from the ileal mucosa with the RNeasy Kit (Accurate Biotechnology [Hunan] Co., Ltd.) in accordance with the instructions provided by the manufacturer. Then, by utilizing a PrimeScript RT reagent kit with a gDNA Eraser (Accurate Biotechnology [Hunan] Co., Ltd.), the RNA was reverse transcribed into cDNA. The SYBR Green Premix Pro Taq HS qPCR Kit (Accurate Biotechnology [Hunan] Co., Ltd.) was used for qRT-PCR, which was carried out with the employment of a LightCycle 480 real-time PCR system (Roche Diagnostics, Germany). The relative expression of each gene in the ileal mucosa was calculated by the 2^−ΔΔCT^ method, with β-actin serving as the internal reference. Appendix Table 1 contains all primer sequences.

### 2.9 Western blotting measurement

The ileum tissue’s total protein was extracted using Radio Immunoprecipitation Assay (Beyotime Institute of Biotechnology), which was then stored at −80°C for further study. Following the measurement of total protein concentration with Bicinchoninic acid assays (Beyotime Institute of Biotechnology), Sodium Dodecyl Sulfate PolyAcrylamide Gel Electrophoresis separation, and transfer to PVDF membranes for western blotting. After being blocked for at least an hour, the membrane was incubated with primary antibodies for an entire night. p-NF-κB p65 (1:500; REF:3033S; Cell Signaling Technology, USA), NF-κB p65 (1:1000; REF:6956S; Cell Signaling Technology, USA), p-IκB (1:500; REF: 9246S; Cell Signaling Technology, USA), IκB (1:1000; REF: 4814S; Cell Signaling Technology, USA), β-actin (1:1000; REF: 4970S; Cell Signaling Technology, USA) were used as primary antibodies. After a 2-h incubation period with the goat anti-mouse or rabbit IgG (H + L) secondary antibody (1:5,000, Abiowell, Hunan, China), imprinting was detected by chemiluminescence. Using Image Lab software, the protein content was normalized to β-actin.

### 2.10 Microbial diversity analysis

The microbial diversity of the colon was investigated following previously described procedures (22). The 16S V3 + V4 specific primers were used to extract and amplify bacterial DNA from colonic contents. Paired-end sequencing was carried out on the Illumina HiSeq 2,500 platform (Illumina, San Diego, California). Using Uchime^1^ and Cutadapt v.1.9.1, raw tags were assembled and filtered to produce clean data. Based on UPARSE (v7.0.1001) (23), sequences were grouped into the same OTU (operational taxonomic units) at a 97% similarity level. Through OTUs (Chao1, Shannon, and Simpson), alpha diversity and richness were calculated to analyze the complexity of species diversity for a sample. The differences in bacterial composition between groups were graphically displayed using the partial least squares discriminant analysis, which was carried out in the R language package “mixOmics.” The PICRUSt (Phylogenetic Investigation of Communities by Reconstruction of Unobserved States) software^2^ was additionally utilized to predict the function of the microflora. The assembled HiSeq sequences obtained in this study were submitted to the NCBI’s Sequence Read Archive (SRA, no. PRJNA899839).

### 2.11 Short-chain fatty acids analysis

The quantitative analysis of SCFAs (short chain fatty acids) was performed by gas chromatography in line with previous research (22). Briefly, the frozen digest was thawed and approximately 1.00 g of the sample was weighed. The samples were thoroughly mixed with ddH_2_O before centrifugation at 10,000 *g* for 10 min to obtain the supernatant. Metaphosphoric acid (25% w/v) was supplemented to the extracts at a ratio of 1:9. The supernatant was subjected to SCFA analysis after centrifugation at 10,000 *g* using an Agilent 7890A (Agilent Technologies, Santa Clara, CA, USA).

### 2.12 Statistical analysis

For comparisons between two groups, the data on the growth performance and diarrhea scores were analyzed using two-tailed t-tests (SPSS Inc., Chicago, IL, USA). The other data were analyzed using two-factor analysis of variance (SPSS Inc., Chicago, IL, USA) for a two × two factorial design with diet (0 or 200 mg/kg β-glucan), immunological challenge (saline or LPS) and their interactions as sources of variation. Tukey’s test was used to evaluate the difference between treatment groups, and *p* < 0.05 was used as the criterion for significance of the difference. 0.05 ≤ *p* < 0.10 was considered a significant trend. Where there was a significant trend for interaction, data were further analyzed using one-way ANOVA with Duncan’s multiple range tests. Correlations between bacterial abundance (at the general level) and proinflammatory cytokine levels in plasma and ileal mucosa SCFA levels were evaluated by Spearman’s correlation test using the R language package ‘Pheatmap.’

## 3 Results

### 3.1 Effect of β-glucan on growth performance and diarrhea in weaned piglets

**Figure 1:**
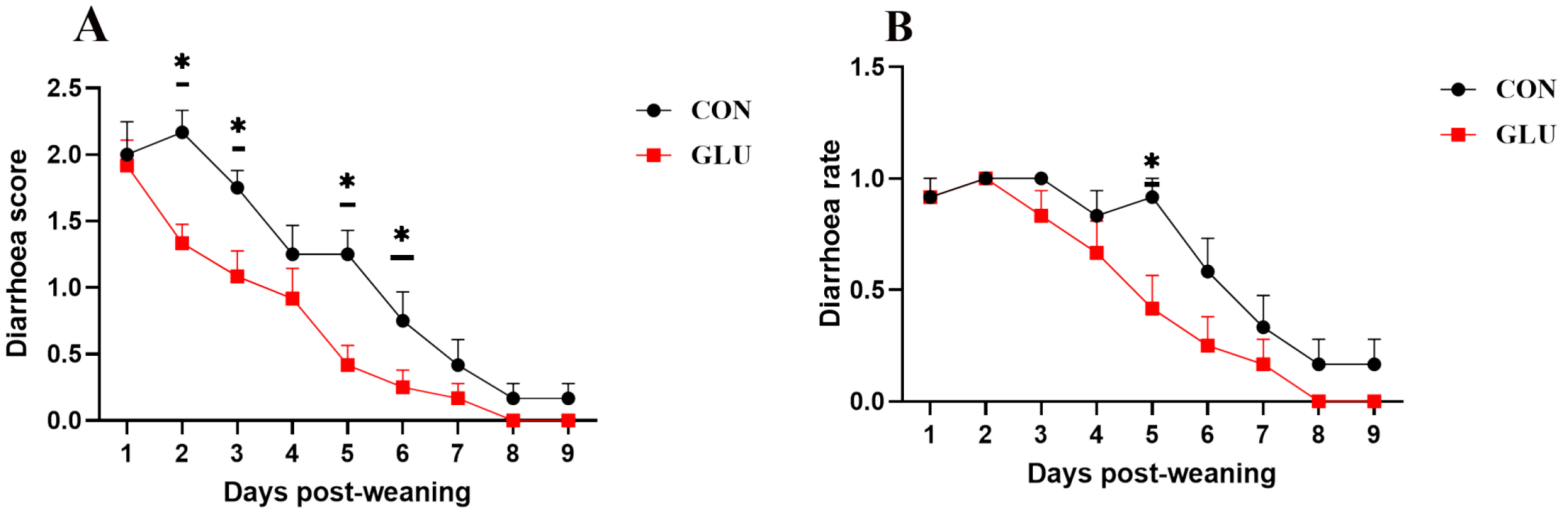
Dietary supplementation with β-glucan alleviate diarrhea of piglet. **(A)** Diarrhea score. **(B)** Diarrhea rate. Data were expressed as means ± SEM (*n* = 12). Significance was presented as *p < 0.05, **p < 0.01, and ***p < 0.001.

There was no significant difference in F:G between the groups from day 1 to 28 (*p* > 0.05). Dietary supplementation with β-glucan significantly increased the final body weight of piglets. Moreover, such supplementation significantly increased the ADG and ADFI of piglets (0.001 < *p* < 0.01; Table 2). In comparison to the control group, the β-glucan group had lower diarrhea scores on days 2, 3, 5 and 6 after weaning, and had lower diarrhea scores and rate on day 5 after weaning (0.01 < *p* < 0.05; Figures 1A,B).

**Table 2:** Dietary supplementation with β-glucan on the growth performance of piglets.

Parameter and period (days)	CON	GLU	SEM	*p*-value
Initial body weight (kg)	6.63	6.65	0.159	0.960
19 Days body weight (kg)	10.60	11.37	0.210	0.067
Final body weight (kg)	19.62	21.54	0.367	0.006
ADG (g/d)				
1–19	208.86	248.20	5.966	0.001
20–39	450.75	508.58	13.054	0.023
1–39	332.91	381.73	8.007	0.001
ADFI (g/d)				
1–19	356.49	424.45	11.222	0.001
20–39	854.40	929.39	30.396	0.225
1–39	608.08	682.51	17.317	0.028
Feed:gain ratio (F/G)				
1–19	1.71	1.72	0.028	0.908
20–39	1.90	1.83	0.050	0.475
1–39	1.83	1.79	0.036	0.568

CON, basic diet without β-glucan; GLU, basic diet with 200 mg/kg β-glucan.

Data were expressed as means (*n* = 12).

### 3.2 Effect of β-glucan on LPS-induced acute inflammation in weaned piglets

**Figure 2:**
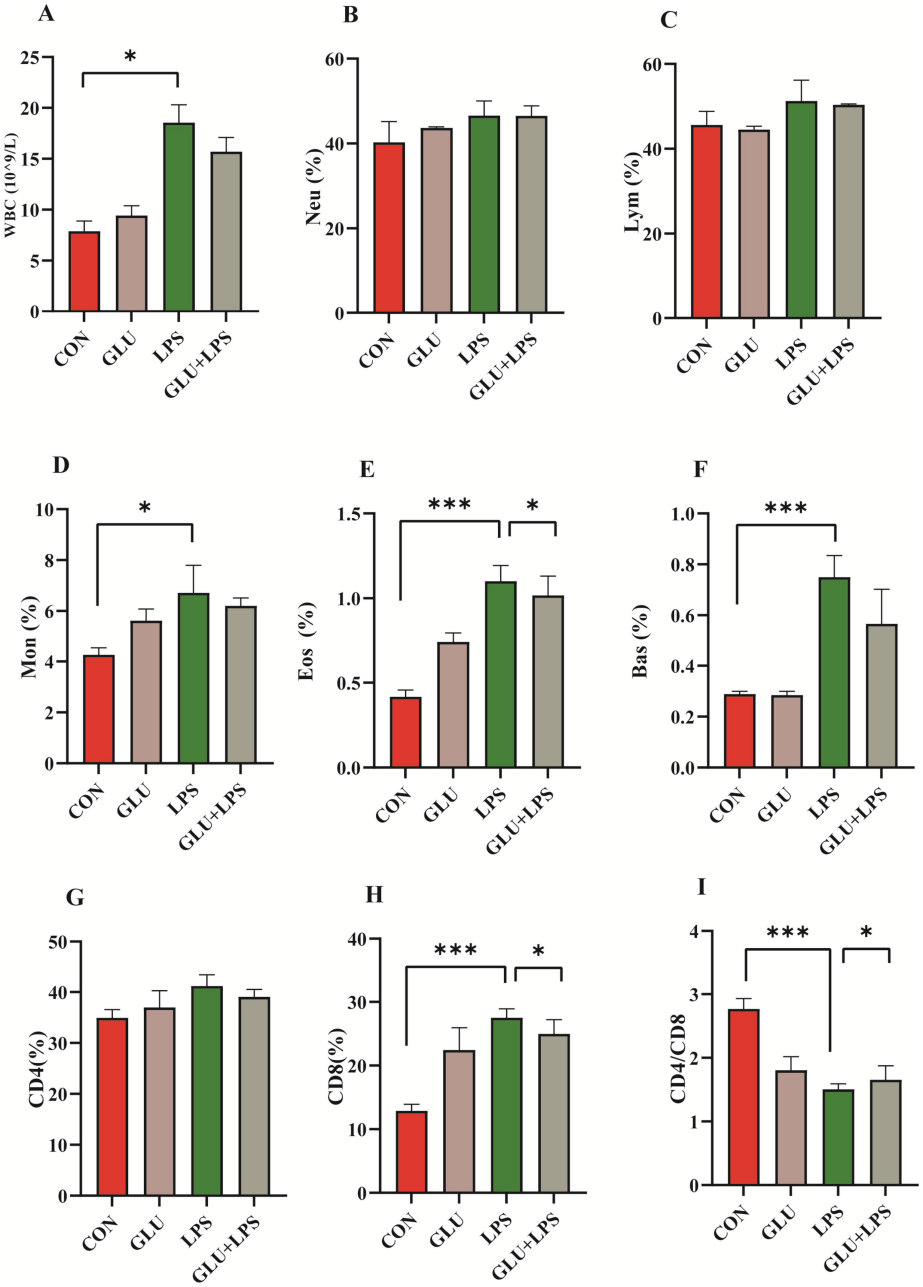
Dietary supplementation with β-glucan alleviate LPS-induced inflammation of piglet. **(A–F)** Blood cell composition. **(G–I)** Lymphocyte subsets composition. Data were expressed as means ± SEM (*n* = 6). Significance was presented as *p < 0.05, **p < 0.01, and ***p < 0.001.

Intestinal inflammation in piglets was induced by intraperitoneal injection of LPS for 4 h, including an increase in leukocytes and the percentage of monocytes, eosinophils, basophils and CD8+ T cells in the whole blood and a decrease in the ratio of CD4+/CD8+ T cells (*p* < 0.05; Figures 2A,D–F,H,I). Dietary supplementation with β-glucan could attenuate the increase in lymphocytes, eosinophils, and CD8+ T cells after the LPS challenge (*p* < 0.001; Figures 2A,E,H).

### 3.3 Effect of β-glucan on intestinal damage in LPS-induced piglets

**Figure 3:**
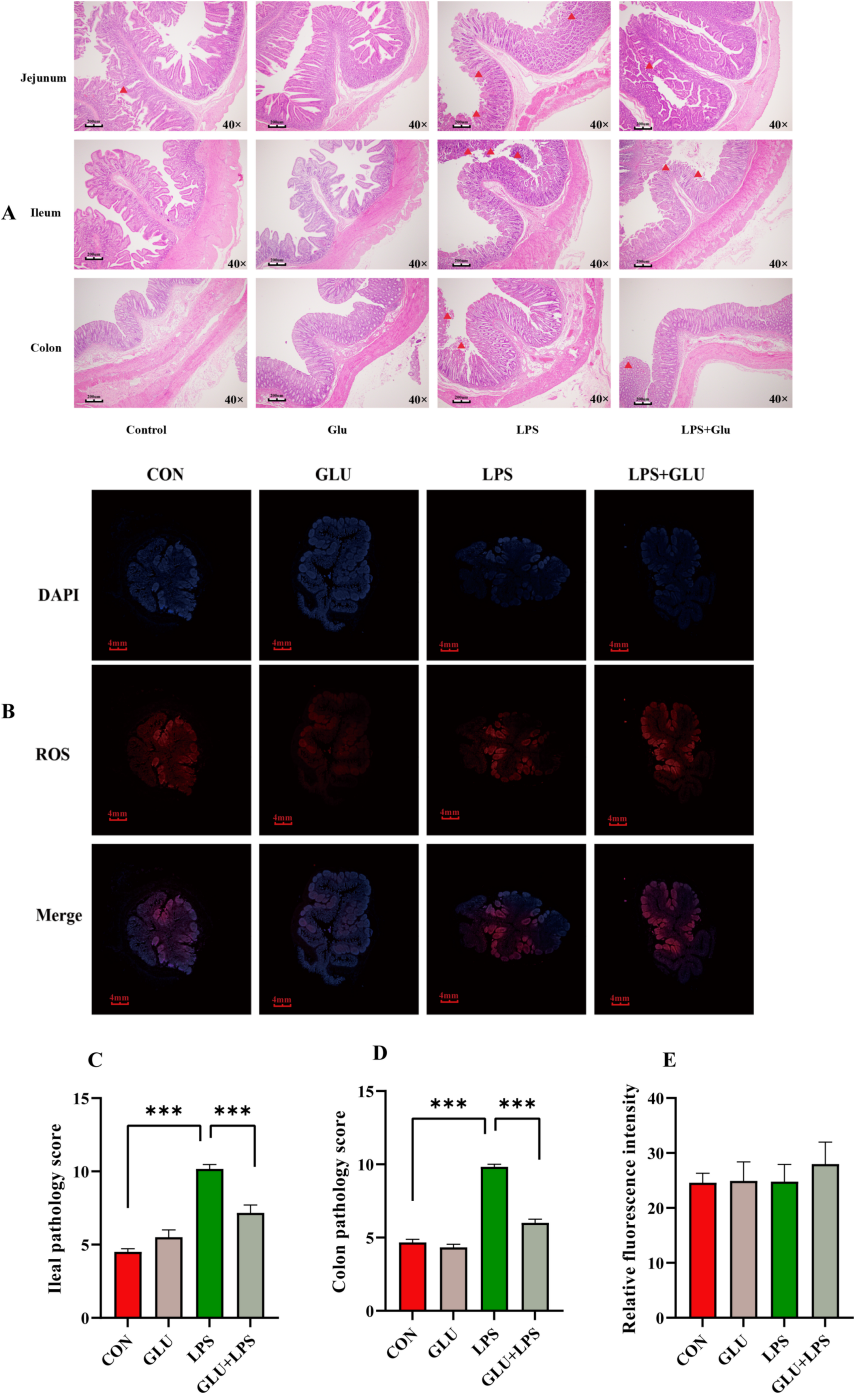
Dietary supplementation with β-glucan alleviate LPS-induced intestinal histological changes of piglet. **(A,C,D)** The H&E staining of the ileal and colon morphology of piglet. **(B,E)** The fluorescent micrographs of ROS (red) and DAPI (blue) staining of ileal. Data were expressed as means ± SEM (*n* = 6). Significance was presented as *p < 0.05, **p < 0.01, and ***p < 0.001.

Ileal and colonic tissue images stained with H&E showed significant tissue damage in LPS-treated piglets, including crypt loss, leukocyte infiltration and intestinal epithelial erosion. However, the addition of β-glucan significantly reduced these signs of damage (Figures 3A,B). LPS significantly increased ileal and colonic histological scores, which were significantly reduced by β-glucan supplementation (*p* < 0.001; Figures 3C,D). LPS and dietary β-glucan supplementation exerted no significant effect on ROS levels (*p* > 0.05; Figure 3E).

### 3.4 Effect of β-glucan on secretion of serum and mucosal cytokines, sIgA, and NO

**Figure 4:**
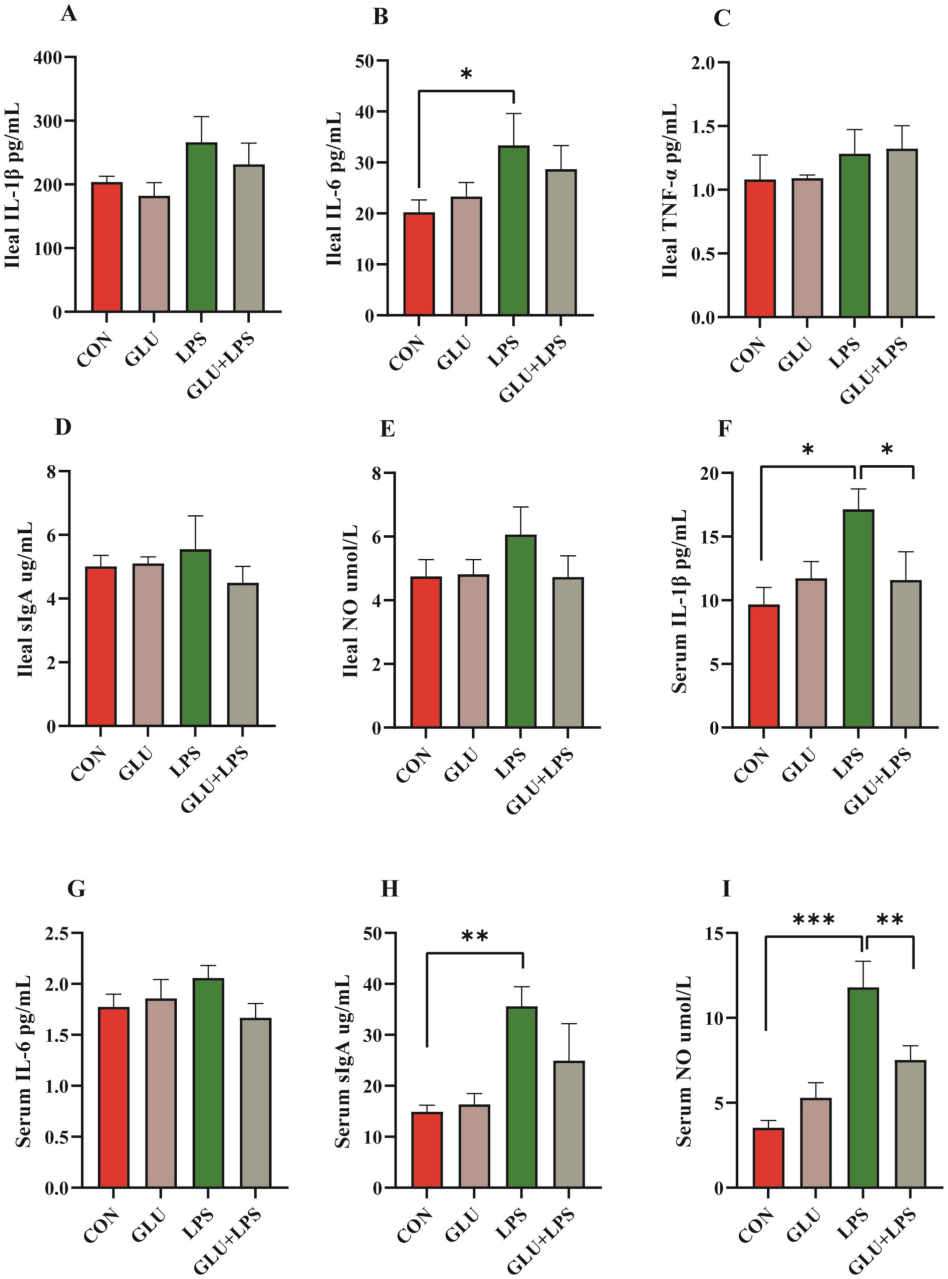
Dietary supplementation with β-glucan decreased the content of pro-inflammatory cytokines and NO of LPS-challenged piglet in plasma and ileal mucosa. **(A–E)** The concentrations of IL-1β, IL-6, TNF-α, sIgA and NO ileal mucosa. **(F–I)** The concentrations of IL-1β, IL-6, sIgA and NO in serum. Data were expressed as means ± SEM (*n* = 6). Significance was presented as *p < 0.05, **p < 0.01, and ***p < 0.001.

LPS treatment significantly increased the levels of the cytokines IL-6 in the ileal mucosa (0.01 < *p* < 0.05; Figure 4B) and IL-1β (0.01 < *p* < 0.05; Figure 4F), sIgA (0.001 < *p* < 0.01; Figure 4H), and NO (*p* < 0.001; Figure 4I) in the serum. β-glucan supplementation significantly rescued the LPS-induced increase in serum IL-1β (0.01 < *p* < 0.05; Figure 4F) and NO levels (0.001 < *p* < 0.01; Figure 4I) and protected the piglet from intestinal inflammation.

### 3.5 Effect of β-glucan on mRNA and protein expression of NF-κB signaling pathway in ileal mucosa

**Figure 5:**
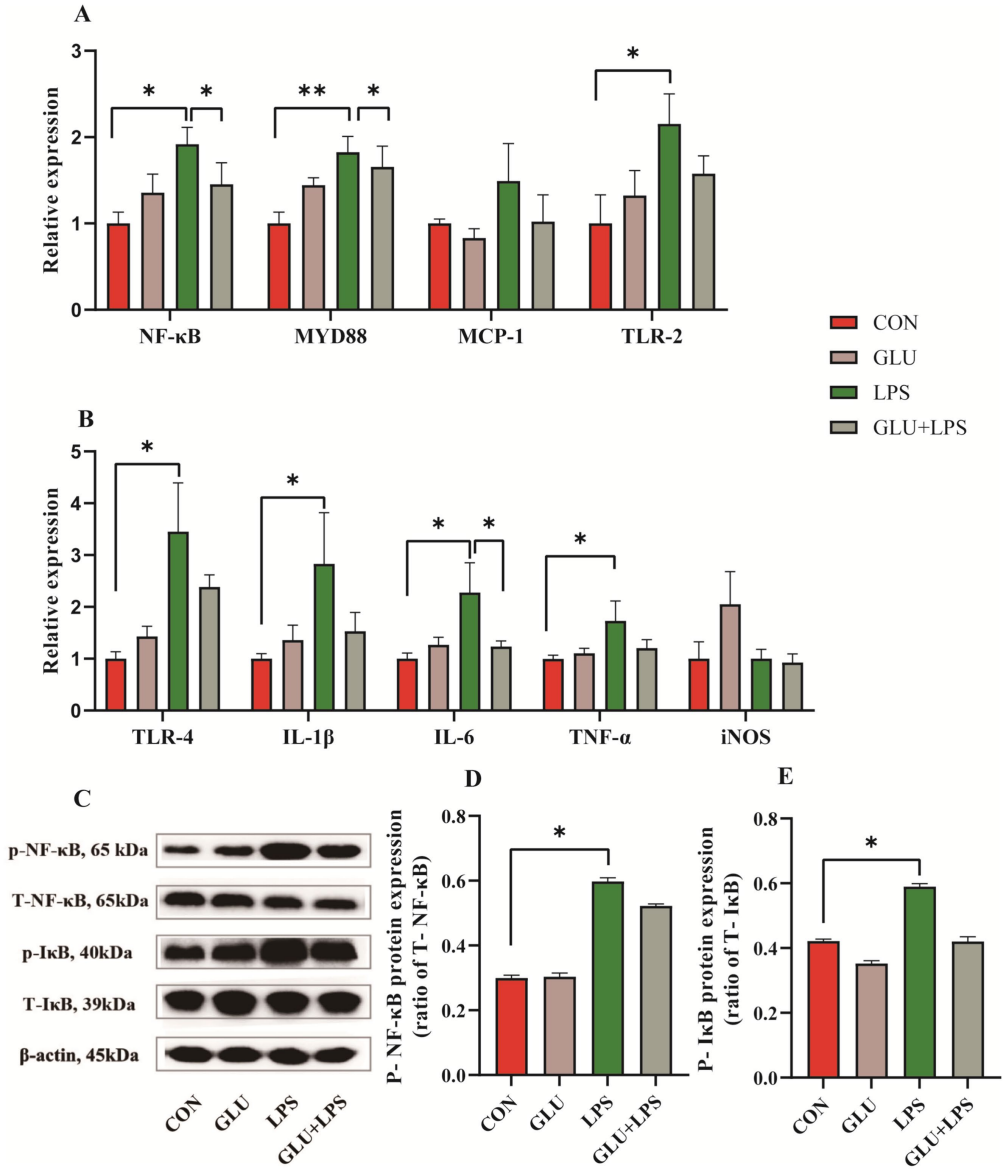
Dietary supplementation with β-glucan inhibited the NF-κB signaling in ileal tissues of piglet. **(A,B)** The mRNA relative expression of *NF-κB, MYD88, MCP-1, TLR-2, TLR-4, IL-1β, IL-6, TNF-α* and *iNOS*. **(C)** Representative protein bands for p-NF-κB, T-NF-κB65, p-IκB, and T-IκB. **(D,E)** Statistical analysis of protein bands. Data were expressed as means ± SEM (*n* = 3). Significance was presented as *p < 0.05, **p < 0.01, and ***p < 0.001.

Treatment with LPS significantly increased the mRNA expression of NF-κB (nuclear factor kappa-B) (0.01 < *p* < 0.05; Figure 5A), MYD88 (myeloid differentiation factor 88) (0.001 < *p* < 0.01; Figure 5A), TLR-2 (Toll-like receptor-2) (0.01 < *p* < 0.05; Figure 5A), IL-1β, IL-6 and TNF-*α* (0.01 < *p* < 0.05; Figure 5B) and the protein expression of phosphorylated NF-κB (0.01 < *p* < 0.05; Figure 5D) and IκB (inhibitor of nuclear factor kappa-B) (0.05 < *p* < 0.1; Figure 5E). The mRNA expression of NF-κB, MYD88 (0.05 < *p* < 0.1; Figure 5A) and IL-6 (0.01 < *p* < 0.05; Figure 5B) of the NF-κB signaling pathway was reduced by supplementation of β-glucan after LPS treatment.

### 3.6 Gut microbiota profiling

**Figure 6:**
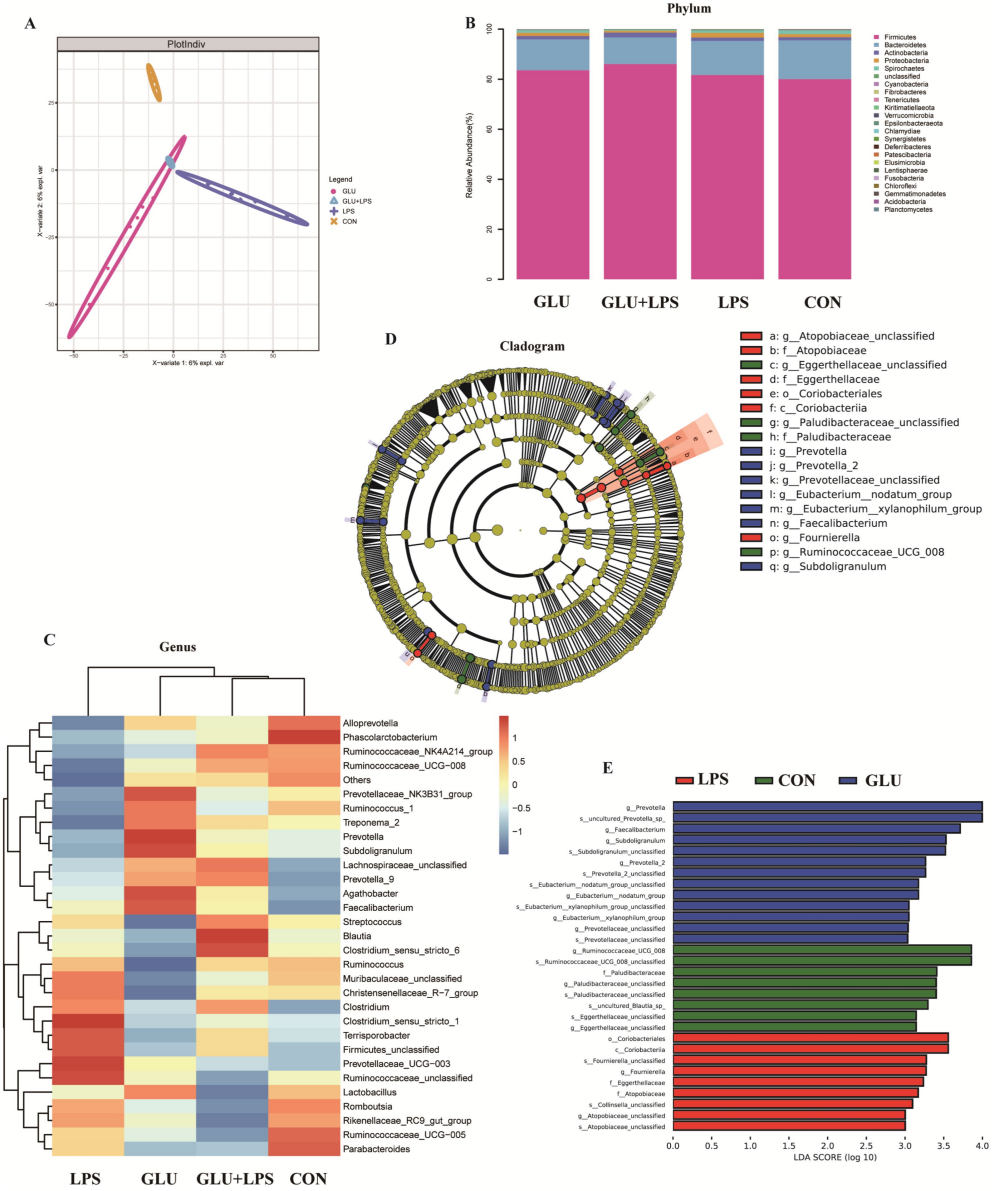
Dietary supplementation with β-glucan altered the composition of the colon microbiota in piglet. **(A)** Partial least squares discriminant analysis (PLS-DA) of gut microbiota. **(B,C)** Relative abundance of predominant bacteria was shown at the phylum and genus. **(D)** Taxonomic cladogram of LEfSe analysis. Different colors indicate the enrichment of the biomarker taxa in the control (green), LPS (red), and Glu (blue) group. The circle from inside to outside means the rank from kingdom to species, and the circle size represents the taxa abundance in the community. **(E)** Linear discriminant analysis (LDA) score for different taxa abundances. Data were expressed as means ± SEM (*n* = 6).

The colonic microbial community of the piglets with different treatments was evaluated by a 16S rDNA phylogenetic method with OTUs >97% similarity. There was no significant difference between dietary β-glucan and LPS treatments in the abundance or uniformity (Appendix Table 2) of the colonic microflora. However, PLS-DA (Partial Least Squares Discriminant Analysis) (Figure 6A) at the OTU level revealed clear segregation and differences in microbiota composition between the four groups. Firmicutes, Bacteroidetes, Actinobacteria, Proteobacteria and Spirochetes were the dominant flora in the colons of piglets in each group. The relative abundance of the bacterial communities was examined at the phylum and genus level (Figure 6C) (Appendix Table 3). Compared to piglets fed a basal diet, β-glucan supplementation reduced the abundance of Synergistetes at the phylum level (0.01 < *p* < 0.05). The top 30 genera were selected for comparison. *Agathobacter* and *Subdoligranulum* had higher abundances after the addition of β-glucan (0.01 < *p* < 0.05). LEfSe (The linear discriminant analysis effect size) method (Figures 6D,E) was used to examine the composition of gut microbes. In the GLU group (β-glucan supplementation group), *Prevotella*, *Faecalibacterium*, *Subdoligranulum*, *Prevotella-2* and *Eubacterium* emerged as dominant species.

### 3.7 Function and metabolism of intestinal microbiota

**Figure 7:**
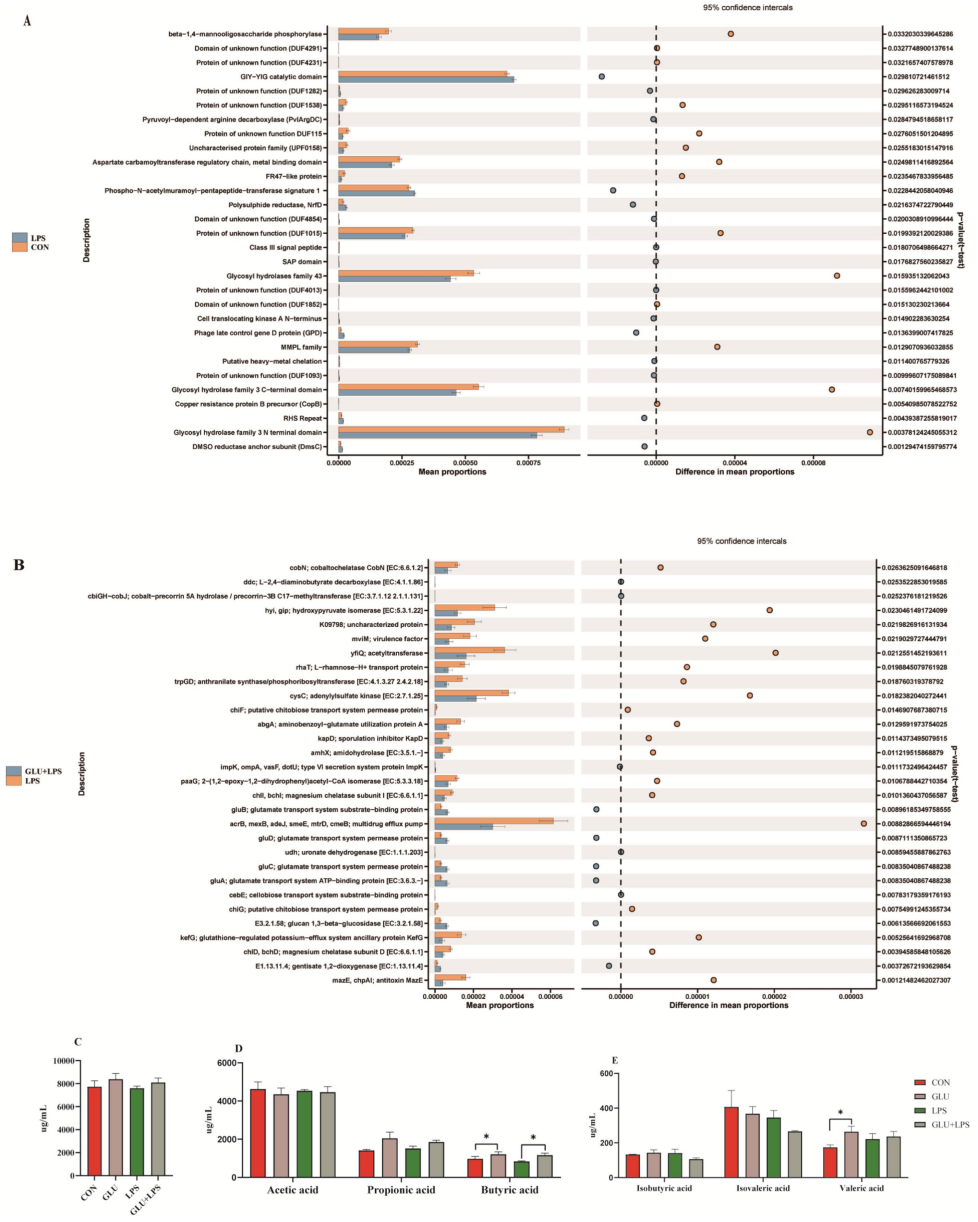
The pathway of different abundances of microflora at STAMP variance analysis **(A,B)** and short-chain fatty acid composition in colon **(C–E)**. Data were expressed as means ± SEM (*n* = 6). Significance was presented as *p < 0.05, **p < 0.01, and ***p < 0.001.

The PICRUST software was used to predict the role of the microbial communities, and the outcomes were compared to recognized metabolic pathways (Figures 7A,B). At KEGG level 3, the function of microbial genes involved in starch and sucrose metabolism, porphyrin and chlorophyll pathways were significantly reduced in the LPS group compared to the control group, while the function of microbial genes involved in steroid hormone biosynthesis was significantly increased. The Glu-LPS group (β-glucan supplementation and LPS treatment group) showed significantly lower gene function in the sugar transport system, phenylalanine, tyrosine and tryptophan biosynthesis and significantly higher gene function in the starch and sucrose metabolism pathways compared to the LPS group. The majority of the alterations were connected to the metabolism of carbon, starch, and sucrose. In addition, dietary β-glucan supplementation increased the levels of butyric acid and valeric acid in the colon, which further validated the above results (Figures 7D,E).

### 3.8 The correlation between the differential bacteria and the examined indices

**Figure 8:**
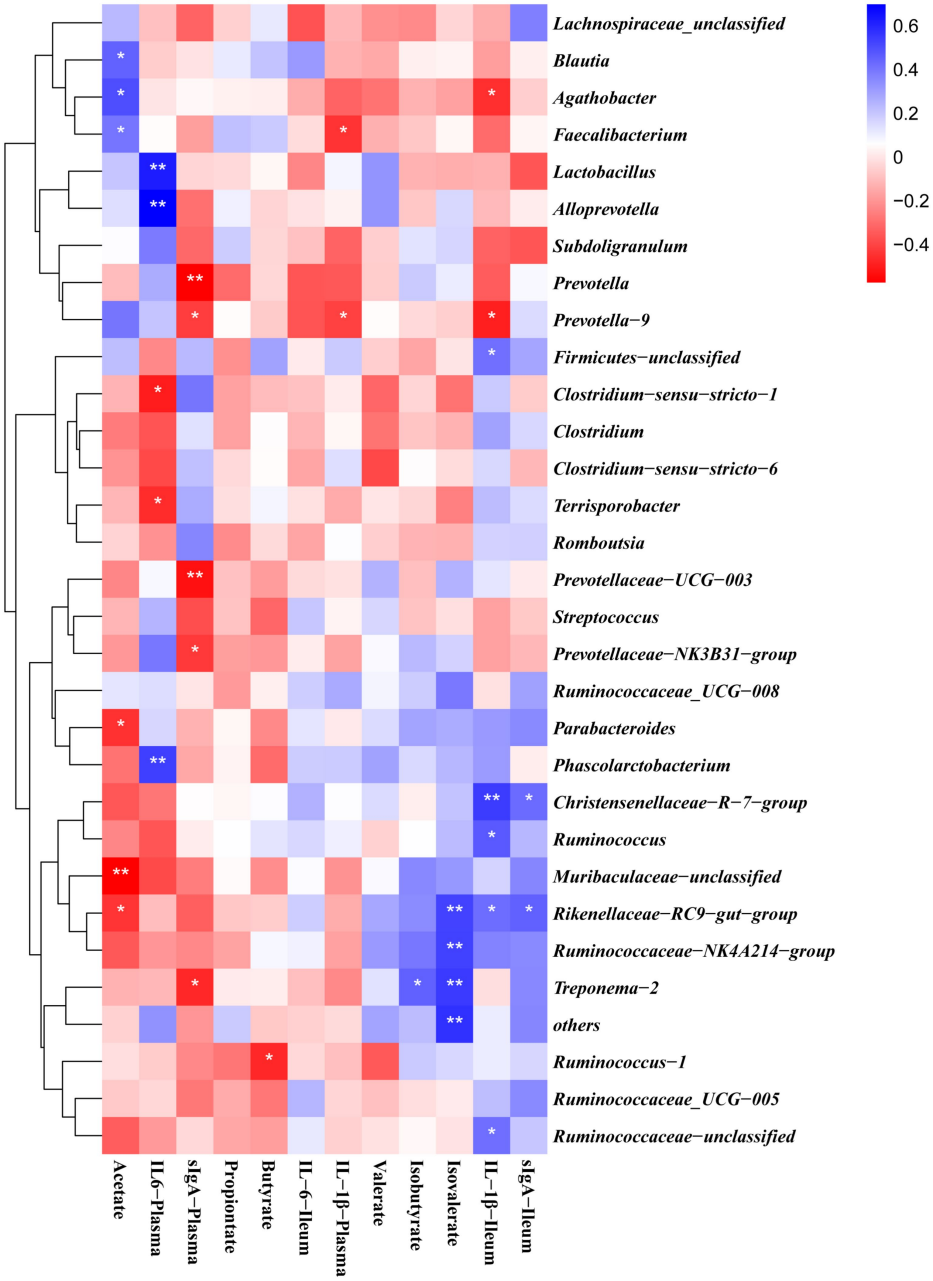
Correlations between bacterial abundance (at the genera level) and pro-inflammatory cytokines contents in plasma and ileal mucosa or colon SCFA content. The red represents a positive correlation, and the blue represents a negative correlation. *and **are used to indicate the statistical significance of the correlation, significance was presented as *p < 0.05, ** *p* < 0.01.

The gut microbiota is involved in the regulation of host immunity. Therefore, the correlation between the levels of pro-inflammatory cytokines in serum and ileal mucosa was analyzed, the levels of SCFA in the colon and the abundance of colonic microorganisms. Spearman correlation analysis (Figure 8) was performed on the gut flora of weaned piglets at the genus level using R software. Acetate content was positively correlated with *Agathobacter*, *Blautia, Prevotella_9* and *Faecalibacterium* and negatively correlated with *Ruminococcacea* and *Muribaculaceae*. Propionate content correlated positively with *Prevotella_9*. Butyrate content was negatively correlated with *Ruminococcus_1*. Isovalerate content was positively correlated with *Ruminococcaceae_NK4A214_group*, *Treponema_2* and *Faecalibacterium*. Plasma IL-6 was positively correlated with *Alloprevotella*, *Phascolarctobacterium*, *Streptococcus* and *Prevotellaceae_NK3B31_group* and negatively correlated with *Clostridium_sensu_stricto_1*. The content of sIgA in ileal mucosa was positively correlated with *Muribaculaceae*, *Ruminococcaceae_UCG-005*, *Rikenellaceae_RC9_gut_group* and *Ruminococcaceae_UCG-008*. IL-1β in ileal mucosa was positively correlated with *Firmicutes*, *Ruminococcaceae_NK4A214_group* and *Christensenellaceae_R-7_group* and negatively correlated with *Prevotella_9*.

## 4 Discussion

The small intestine is the main digestive and absorptive organ, as well as the largest immune and barrier organ in the body (24). The results of this study indicate that feeding β-glucan can enhance piglet immunity, alleviate intestinal damage after LPS challenge, and promote piglet growth through modulating gut microbiota composition and metabolism. Intraperitoneal injection of LPS, which is commonly used to induce intestinal inflammation in piglets (25, 26), and the composition of lymphocyte subsets, often regarded as an effective indicator to determine whether the body is producing inflammation (27). In this study, LPS was injected intraperitoneally to induce intestinal inflammation in piglets. The results showed a significant decrease in the CD4^+^/CD8^+^T cell ratio and a significant increase in the number of neutrophils and lymphocytes in the blood of piglets, indicating the successful establishment of a piglet inflammation model.

Intestinal morphology is one of the key indicators of intestinal health (28). LPS challenge can induce morphological changes in the intestine, including villus shedding, vacuolization and necrosis of intestinal epithelial cells (20, 29, 30). In this study, LPS caused intestinal epithelial erosion, villous atrophy, and monocyte and lymphocyte infiltration in the ileum and colon of piglets, but the addition of β-glucan reduced the histopathological scores of the ileum and colon tissues in piglets. Cytokines IL-1 β, IL-6, and TNF-*α* are involved in the occurrence of intestinal inflammation (31). Previous studies have shown that supplementation of feed with β-glucan reduces the TNF-*α* concentration in plasma of LPS-attacked piglets (32). Our current study revealed that LPS challenge significantly increased the levels of IL-6 in the ileal mucosa and IL-1 β in serum of piglets, whereas supplementation with β-glucan reduced the levels of IL-1 β in serum. In addition, NO produced by the body under normal conditions activates protective inflammatory pathways, which regulates the host response to exogenous pathogens, but overproduction of NO leads to its accumulation under abnormal conditions and can induce oxidative stress (33). In the current study, serum NO levels were significantly higher in LPS-treated piglets, whereas β-glucan significantly reduced serum NO levels. These results suggest that glucan may inhibit intestinal inflammation by mediating inflammatory factors.

The TLR/NF-κB signaling pathway is crucial for both innate and adaptive immune responses, and NF-κB is a major target of the inflammatory response (34). Activation of Toll like receptors leads to the production of IL-1 β, IL-6, and TNF-α, which participate in the immune response of bacteria and pathogens (35, 36). Studies have shown that pectin supplementation attenuates endotoxin attacks by inhibiting Toll-like receptor activation (20, 37). This study indicates that LPS exposure increased the mRNA expression levels of NF-κB, TLR-2, TLR-4, MYD88, IL-1β, IL-6 and TNF-α in piglet ileal mucosa as well as the protein expression levels of p-NF-κB and p-IκB in ileal tissue. Dietary supplementation with β-glucan reduced the mRNA expression of NF-κB and inflammatory cytokines, while having no effect on NF-κB protein expression. This might be attributed to the fact that β-glucan could reduce NF-κB mRNA expression either by inhibiting the DNA-binding activity of NF-κB or by inhibiting IKKbeta kinase activity (38, 39). However, due to the influence of post-translational transcriptional regulatory mechanisms of proteins or stress-induced compensatory mechanisms, β-glucan reduced the LPS-induced NF-κB mRNA expression but had no significant effect on protein levels (40, 41). This result is consistent with previous reports in that β-glucan supplementation effectively suppressed the elevated TLR-4 mRNA expression caused by LPS treatment (42). These findings suggest that β-glucan may inhibit LPS-induced inflammation by suppressing TLR/NF-κB activation.

Microorganisms mediate gastrointestinal metabolism, mucosal inflammation, and immune processes in the body, affecting gastrointestinal diseases such as inflammatory bowel disease and colorectal cancer (2, 43, 44). Indeed, the ecological imbalance of gut microbiota can lead to host immune dysfunction, and regulating the composition of gut microbiota can affect gut immunity (10). Some studies have shown that β-glucan feeding alters the cecal microbiota of rats by increasing the abundance of *Bifidobacteria* and *Lactobacillus* (32). Hence, we hypothesized that β-glucan supplementation may influence the microbiota composition in the piglet colon following LPS challenge. 16S rRNA analysis of colonic digest revealed that piglets administered with β-glucan had significantly altered gastrointestinal microbiota after LPS treatment, according to the current study. LPS challenge reduced the abundance of *Ruminococcus_1* and *Subdoligranulum*, whereas β-glucan increased the relative abundance of beneficial bacteria such as *Prevotella*, *Agathobacter*, *Faecalibacterium*, *Prevotella_9* and *Subdoligranulum*. β-glucan supplementation restored the colonic microbial community after LPS challenge.

We used PICRUST2 to examine the functional characteristics of the gut microbiota. The LPS challenge significantly lowered the functions of starch and sucrose metabolism, porphyrin and chlorophyll metabolism, and manno-oligosaccharide phosphorylation. In contrast, supplementing with β-glucan significantly improved the processes of tyrosine, starch, and sucrose metabolism. Thus, β-glucan supplementation may aid in regulating abnormal intestinal flora function brought on by LPS and maintaining intestinal homeostasis during weaning. In addition, we discovered that the most of the characteristically predicted changes in biological functions were associated with sugar metabolism, including the metabolism of starch, sucrose, and carbon. There was a significant increase in propionate and butyrate concentrations after β-glucan supplementation, which could be due to β-glucan promoting the development of microbial fermentation processes associated with an increased abundance of certain butyrate-producing bacteria, such as *Subdoligranulum* and *Prevotella-9*. Previous studies have shown that SCFAs can promote the maturation of the gastrointestinal tract, improve intestinal barrier function, and regulate body immunity, thereby reducing diarrhea rates and improving piglet growth performance (45–47). Correlation analysis revealed that the impact of β-glucan on the gut flora of piglets was strongly related to changes in the composition of SCFAs and inflammatory cytokines, and that enhancing the composition of the gut flora and its metabolites improved the gut function and growth performance of the host.

These findings indicate that β-glucan can enhance piglet immunity, alleviate intestinal damage after LPS challenge, and promote piglet growth by affecting gut microbiota composition and metabolism.

## Data Availability

The datasets presented in this study can be found in online repositories. The names of the repository/repositories and accession number(s) can be found in the article/Supplementary material.

## Footnotes

^1^ http://www.drive5.com/usearch/manual/uchime_algo.html

^2^ http://picrust.github.com
