# Supplementary material for: The effect of a water-soluble β-glucan on intestinal immunity and microbiota in LPS-challenged piglets
Source: Front Vet Sci. 2025 Mar 10;12:1533872. doi: 10.3389/fvets.2025.1533872 (PMC11931652; doi:10.3389/fvets.2025.1533872)
Supplement: Supplementary file 1 [file Supplementary_file_1.docx]

***Supplementary Material***

The effect of a water-soluble β - glucan on intestinal immunity and microbiota in LPS-challenged piglets

**Yuliang Wu^1,2^,** **Yuxin Li^1,2^, Mengli Chen^1,2^, Juan Zhao^3^,** **Xia Xiong^2,4,5*^, Chen Guang Olnood^3^,** **Yundi Gao^3^,** **Fei Wang^3^,** **Can Peng^2,4^,** **Miao Liu^6,7^,** **Chunxia Huang^5^,** **Jianzhong Li^1^,** **Liuqin He^1^, Huansheng Yang^1*^,** **Yulong Yin ^1,2,4^**

^1^Laboratory of Animal Nutrition and Human Health, College of Life Sciences, Hunan Normal University, Changsha 410081, China

^2^CAS Key Laboratory of Agro-ecological Processes in Subtropical Region, Institute of Subtropical Agriculture; Hunan Province Key Laboratory of Animal Nutritional Physiology and Metabolic Process, National Engineering Laboratory for Pollution Control and Waste Utilization in Livestock and Poultry Production, Institute of Subtropical Agriculture, Chinese Academy of Sciences, Changsha 410125, China

^3^Sichuan Synlight Biotech Ltd., Chengdu, 610041, China

^4^University of Chinese Academy of Sciences, Beijing, China

^5^Changsha Medical University, Changsha, China

^6^Key Laboratory of Mollisols Agroecology, Northeast Institute of Geography and Agroecology, Chinese Academy of Sciences, Changchun 130102, China

^7^Jilin Da’an Agro-ecosystem National Observation Research Station

* **Correspondence author：**

E-mail addresses: xx@isa.ac.cn (X. Xiong), yhs@hunnu.edu.cn (H. S. Yang)

**Appendix Figure 1.** **Flowchart of experimental design.**

**
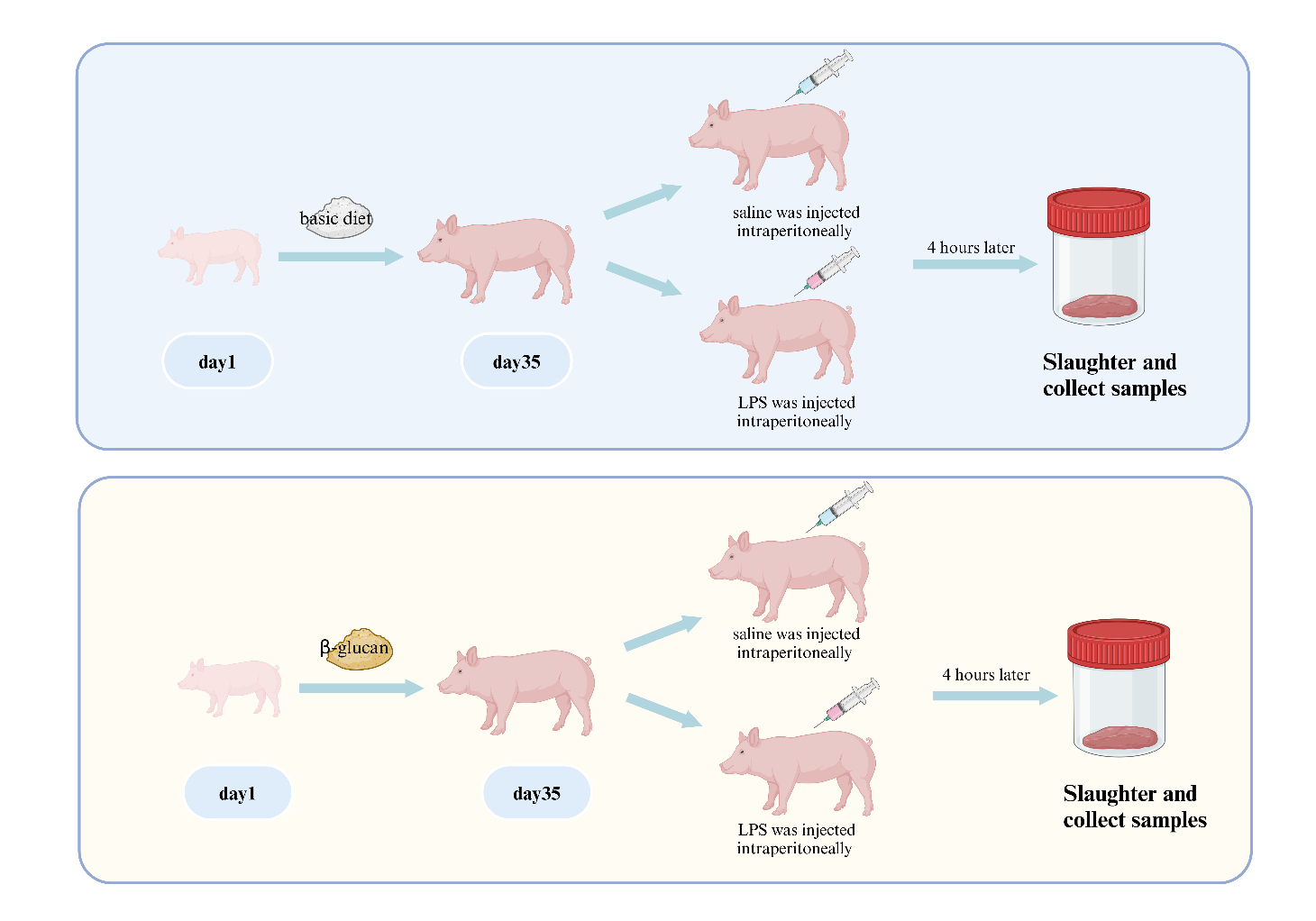
**

**Appendix Table 1.** **Primer pairs used for the Quantitative real-time PCR.**

| **Gene** | **GeneBank no.** | **Sequence (5'–3')** | **Product length, bp** |
| --- | --- | --- | --- |
| *β-actin* | XM_003124280.5 | F: CTGCGGCATCCACGAAACT | 147 |
|  |  | R: AGGGCCGTGATCTCCTTCTG |  |
| *IL-1β* | NM_214055.1 | F: ACGTGCAATGATGACTTTGTCTG | 113 |
|  |  | R: AGAGCCTTCAGCATGTGTGG |  |
| *TNF-α* | NM_214022.1 | F: CATCGCCGTCTCCTACCA | 199 |
|  |  | R: CCCAGATTCAGCAAAGTCCA |  |
| *IL-6* | NM_214399.1 | F: CCCACCAGGAACGAAAGAGA | 82 |
|  |  | R: CTGGACTGAAGGCGCTTGT |  |
| *NF-kB* | XM_013988356.2 | F: CAGCTCTCACAACCTCACCA | 180 |
|  |  | R: GTGATCCAGTTAGGGGGACA |  |
| *MYD88* | NM_001099923.1 | F: TGATGCCTTCATCTGCTACTG | 109 |
|  |  | R: ATCTCGGTCAGACACACATAAC |  |
| *TLR-2* | NM_213761.1 | F: CGGTGTGCTGCAAGGTCAAC | 133 |
|  |  | R: CAGCAGGGTCACAAGACAGA |  |
| *TLR-4* | NM_001293316.1 | F: CTGCCTTCACTACAGAGACTTC | 102 |
|  |  | R: TGGGACACCACGACAATAAC |  |
| *MCP-1* | NM_214214.1 | F: CTTGCCCAGCCAGATGCAAT | 113 |
|  |  | R: CACTTGCTGCTGGTGACTCT |  |
| *iNOS* | XM_013981169.2 | F: GCTGTCGTGGAGATCAATGT | 123 |
|  |  | R: GCTGTCGTGGAGATCAATGT |  |

**Appendix Table 2.** **Bacterial community diversity in colon of piglet.**

|  | CON | | GLU | | SEM | | P-Value | | |
| --- | --- | --- | --- | --- | --- | --- | --- | --- | --- |
| Item | Saline | LPS | Saline | LPS |  |  | diet | LPS | Diet×LPS |
| OTU | 899.83 | 908.50 | 883.50 | 775.17 | | 39.25 | 0.365 | 0.544 | 0.477 |
| Chao1 | 908.37 | 920.93 | 893.75 | 790.50 | | 39.81 | 0.388 | 0.587 | 0.489 |
| shannon | 7.18 | 7.01 | 7.06 | 6.35 | | 0.15 | 0.182 | 0.13 | 0.352 |
| Simpson | 0.96 | 0.96 | 0.96 | 0.92 | | 0.01 | 0.162 | 0.162 | 0.162 |

**Appendix Table 3.** **Composition of colonic bacteria in piglets in terms of genus and species**

|  | CON | | GLU | | SEM | P-Value | | |
| --- | --- | --- | --- | --- | --- | --- | --- | --- |
| Item | Saline | LPS | Saline | LPS |  | Diet | LPS | Diet×LPS |
| Phylum |  |  |  |  |  |  |  |  |
| Firmicutes | 80.02 | 81.69 | 83.56 | 86.11 | 1.440 | 0.193 | 0.481 | 0.883 |
| Bacteroidetes | 15.52 | 13.65 | 12.41 | 10.61 | 1.218 | 0.236 | 0.474 | 0.989 |
| Actinobacteria | 1.23 | 1.31 | 1.30 | 1.94 | 0.121 | 0.137 | 0.123 | 0.224 |
| Proteobacteria | 1.25 | 1.88 | 1.28 | 0.73 | 0.231 | 0.244 | 0.934 | 0.219 |
| Spirochaetes | 1.51 | 0.89 | 0.98 | 0.36 | 0.209 | 0.212 | 0.147 | 0.992 |
| unclassified | 0.11 | 0.27 | 0.09 | 0.20 | 0.050 | 0.672 | 0.201 | 0.817 |
| Cyanobacteria | 0.12 | 0.07 | 0.08 | 0.01 | 0.020 | 0.225 | 0.137 | 0.881 |
| Fibrobacteres | 0.06 | 0.04 | 0.09 | 0.00 | 0.017 | 0.952 | 0.119 | 0.263 |
| Tenericutes | 0.05 | 0.06 | 0.04 | 0.01 | 0.011 | 0.106 | 0.736 | 0.319 |
| Kiritimatiellaeota | 0.05 | 0.03 | 0.01 | 0.00 | 0.012 | 0.153 | 0.624 | 0.806 |
| Verrucomicrobia | 0.00 | 0.01 | 0.06 | 0.01 | 0.013 | 0.344 | 0.464 | 0.277 |
| Epsilonbacteraeota | 0.02 | 0.02 | 0.02 | 0.00 | 0.003 | 0.201 | 0.201 | 0.662 |
| Chlamydiae | 0.00 | 0.00 | 0.05 | 0.00 | 0.012 | 0.352 | 0.352 | 0.314 |
| Synergistetes | 0.01 | 0.03 | 0.00 | 0.00 | 0.004 | 0.024 | 0.420 | 0.420 |
| Deferribacteres | 0.01 | 0.03 | 0.01 | 0.00 | 0.006 | 0.295 | 0.545 | 0.234 |
| Patescibacteria | 0.02 | 0.00 | 0.01 | 0.01 | 0.004 | 0.681 | 0.145 | 0.496 |
| Elusimicrobia | 0.01 | 0.01 | 0.01 | 0.00 | 0.003 | 0.854 | 0.363 | 0.208 |
| Fusobacteria | 0.01 | 0.00 | 0.00 | 0.00 | 0.002 | 0.153 | 0.332 | 0.332 |
| Lentisphaerae | 0.00 | 0.00 | 0.00 | 0.01 | 0.001 | 0.238 | 0.689 | 0.238 |
| Chloroflexi | 0.00 | 0.00 | 0.00 | 0.00 | 0.001 | 0.198 | 0.661 | 0.661 |
| Gemmatimonadetes | 0.00 | 0.00 | 0.00 | 0.00 | 0.001 | 0.332 | 0.332 | 0.332 |
| Acidobacteria | 0.00 | 0.00 | 0.00 | 0.00 | 0.000 | - | - | - |
| Planctomycetes | 0.00 | 0.00 | 0.00 | 0.00 | 0.000 | - | - | - |
| Genus |  |  |  |  |  |  |  |  |
| Clostridium_sensu_stricto_1 | 25.68 | 31.34 | 25.25 | 26.40 | 2.468 | 0.615 | 0.525 | 0.672 |
| Terrisporobacter | 8.94 | 10.95 | 8.57 | 9.88 | 0.897 | 0.710 | 0.395 | 0.853 |
| Streptococcus | 6.15 | 6.61 | 3.87 | 8.04 | 1.660 | 0.905 | 0.522 | 0.608 |
| Lachnospiraceae_unclassified | 3.07 | 3.31 | 4.40 | 4.78 | 0.424 | 0.120 | 0.721 | 0.937 |
| Ruminococcaceae_UCG-005 | 4.61 | 3.89 | 3.27 | 2.80 | 0.381 | 0.127 | 0.445 | 0.870 |
| Romboutsia | 3.45 | 3.28 | 2.37 | 1.91 | 0.386 | 0.136 | 0.691 | 0.853 |
| Prevotella | 1.96 | 1.65 | 3.68 | 2.15 | 0.321 | 0.074 | 0.132 | 0.312 |
| Prevotellaceae_NK3B31_group | 2.33 | 1.69 | 3.15 | 2.07 | 0.368 | 0.440 | 0.275 | 0.776 |
| Muribaculaceae_unclassified | 2.10 | 2.33 | 1.39 | 1.73 | 0.247 | 0.213 | 0.576 | 0.915 |
| Blautia | 1.73 | 1.73 | 1.61 | 2.03 | 0.213 | 0.840 | 0.646 | 0.652 |
| Lactobacillus | 2.25 | 1.19 | 3.01 | 0.43 | 0.602 | 1.000 | 0.156 | 0.541 |
| Clostridium | 1.34 | 1.49 | 1.37 | 1.48 | 0.137 | 0.968 | 0.657 | 0.942 |
| Agathobacter | 0.34 | 0.61 | 3.49 | 1.07 | 0.449 | 0.029 | 0.173 | 0.093 |
| Ruminococcaceae_UCG-008 | 1.81 | 0.60 | 1.10 | 1.73 | 0.266 | 0.696 | 0.582 | 0.100 |
| Rikenellaceae_RC9_gut_group | 1.45 | 1.44 | 1.19 | 0.95 | 0.194 | 0.372 | 0.770 | 0.781 |
| Christensenellaceae_R-7_group | 1.13 | 1.53 | 0.60 | 1.09 | 0.187 | 0.217 | 0.251 | 0.906 |
| Firmicutes_unclassified | 0.90 | 1.28 | 0.90 | 1.09 | 0.134 | 0.745 | 0.319 | 0.740 |
| Treponema_2 | 0.80 | 0.35 | 1.48 | 0.97 | 0.204 | 0.122 | 0.241 | 0.937 |
| Clostridium_sensu_stricto_6 | 0.85 | 0.85 | 0.67 | 1.18 | 0.153 | 0.826 | 0.445 | 0.445 |
| Ruminococcus | 1.08 | 1.08 | 0.33 | 0.95 | 0.234 | 0.378 | 0.535 | 0.532 |
| Prevotellaceae_UCG-003 | 0.76 | 1.01 | 0.86 | 0.79 | 0.105 | 0.768 | 0.695 | 0.486 |
| Ruminococcus_1 | 0.82 | 0.54 | 0.91 | 0.63 | 0.079 | 0.549 | 0.093 | 0.995 |
| Faecalibacterium | 0.35 | 0.60 | 1.22 | 0.66 | 0.139 | 0.090 | 0.547 | 0.138 |
| Prevotella_9 | 0.16 | 0.28 | 1.06 | 1.28 | 0.251 | 0.070 | 0.731 | 0.912 |
| Parabacteroides | 0.84 | 0.71 | 0.54 | 0.54 | 0.084 | 0.196 | 0.715 | 0.690 |
| Alloprevotella | 0.91 | 0.36 | 0.69 | 0.55 | 0.113 | 0.947 | 0.138 | 0.373 |
| Ruminococcaceae_unclassified | 0.59 | 0.70 | 0.59 | 0.53 | 0.050 | 0.423 | 0.821 | 0.434 |
| Subdoligranulum | 0.45 | 0.34 | 1.00 | 0.55 | 0.089 | 0.019 | 0.077 | 0.264 |
| Ruminococcaceae_NK4A214_group | 0.61 | 0.49 | 0.51 | 0.62 | 0.080 | 0.909 | 0.982 | 0.509 |
| others | 22.55 | 17.75 | 20.91 | 21.10 | 1.035 | 0.685 | 0.285 | 0.248 |
